# Gene and protein expression of glucose transporter 1 and glucose transporter 3 in human laryngeal cancer—the relationship with regulatory hypoxia-inducible factor-1α expression, tumor invasiveness, and patient prognosis

**DOI:** 10.1007/s13277-014-2838-4

**Published:** 2014-11-21

**Authors:** Katarzyna Starska, Ewa Forma, Paweł Jóźwiak, Magdalena Bryś, Iwona Lewy-Trenda, Ewa Brzezińska-Błaszczyk, Anna Krześlak

**Affiliations:** 1 I Department of Otolaryngology and Laryngological Oncology, Medical University of Łódź, Kopcinskiego 22, 90-153 Łódź, Poland; 2 Department of Cytobiochemistry, University of Łódź, Pomorska 142/143, 90-236 Łódź, Poland; 3 Department of Pathology, Medical University of Łódź, Pomorska 251, 92-213 Łódź, Poland; 4 Department of Experimental Immunology, Medical University of Łódź, Pomorska 251, 92-213 Łódź, Poland

**Keywords:** *SLC2A1* and *SLC2A3* genes, *HIF-1α* gene, GLUT1 and GLUT3 proteins, HIF-1α protein, Tumor front grading (TFG), Laryngeal cancer

## Abstract

Increased glucose uptake mediated by glucose transporters and reliance on glycolysis are common features of malignant cells. Hypoxia-inducible factor-1α supports the adaptation of hypoxic cells by inducing genes related to glucose metabolism. The contribution of glucose transporter (GLUT) and hypoxia-inducible factor-1α (HIF-1α) activity to tumor behavior and their prognostic value in head and neck cancers remains unclear. The aim of this study was to examine the predictive value of GLUT1, GLUT3, and HIF-1α messenger RNA (mRNA)/protein expression as markers of tumor aggressiveness and prognosis in laryngeal cancer. The level of hypoxia/metabolic marker genes was determined in 106 squamous cell laryngeal cancer (SCC) and 73 noncancerous matched mucosa (NCM) controls using quantitative real-time PCR. The related protein levels were analyzed by Western blot. Positive expression of *SLC2A1*, *SLC2A3*, and *HIF-1α* genes was noted in 83.9, 82.1, and 71.7 % of SCC specimens and in 34.4, 59.4, and 62.5 % of laryngeal cancer samples. Higher levels of mRNA/protein for GLUT1 and HIF-1α were noted in SCC compared to NCM (*p* < 0.05). *SLC2A1* was found to have a positive relationship with grade, tumor front grading (TFG) score, and depth and mode of invasion (*p* < 0.05). *SLC2A3* was related to grade and invasion type (*p* < 0.05). There were also relationships of *HIF-1α* with pTNM, TFG scale, invasion depth and mode, tumor recurrences, and overall survival (*p* < 0.05). In addition, more advanced tumors were found to be more likely to demonstrate positive expression of these proteins. In conclusion, the hypoxia/metabolic markers studied could be used as molecular markers of tumor invasiveness in laryngeal cancer.

## Introduction

Over the past decade, research has been focused on the mechanisms through which various types of neoplasms might take a more malignant phenotype and form more aggressive cell clones which can determine tumor development and progression. It is well known that the rapid proliferation of malignant cells causes the formation of hypoxic areas within the stroma of solid human tumors [1]. Hypoxia is able to induce many changes in the physiological processes and biochemistry of malignant cells by upregulating the expression of a number of target genes, *inter alia* those related to glucose metabolism and glucose transport, angiogenesis, invasion, and metastasis, in order to adapt to anaerobic conditions [1–5]. The results of many studies have provided evidence of the carcinogenic effects of the inhibition of the oxidative phosphorylation process and conversion into enhanced aerobic glycolysis, known as the Warburg effect, which is associated with the activity of key factors in glucose metabolism like glucose transporters (GLUTs) and their most important regulatory hypoxia-related protein, hypoxia-inducible factor-1α (HIF-1α) [6].

Until now, 14 members of the GLUT family coded by genes belonging to the solute carrier 2A family (*SLC2A*) have been identified [7]. Numerous in vivo and in vitro studies suggest that the biological activity of GLUT isoforms and their participation in many regulatory mechanisms involving, among others, the phosphatidylinositol-3-kinase (PI3K)/Akt pathway, NF-κB activity, and wild-type p53 protein expression may contribute to cancer development [8–11]. GLUTs also promote the epithelial-mesenchymal transition (EMT), cell migration, invasion, and metastasis by the regulation of matrix metalloproteinase (MMP) activity [12]. Other mechanisms of carcinogenesis are HIF-1α activity in EMT via the inhibition of E-cadherin and promotion of MMP-2 and SNAI1 expression, the regulation of the PI3K/Akt pathway, as well as VEGF activation [3, 4, 13, 14].

A literature review reveals the contribution of aberrant various glucose uptake transporter isoforms and metabolism/hypoxia regulators to the tumorigenesis and progression of various head and neck cancers [8, 15–22]. Unfortunately, the clinical importance of alternations in glucose metabolism and GLUT expression in these malignances is largely unknown. Most reviews focus on the association between cellular level of GLUTs and both tumor aggressiveness and disease outcome [8, 15–22]. Unfortunately, other studies have reported not only contradictory results and inverse interconnections, but also a lack of any association between the expression of metabolism/hypoxia endogenous markers with either clinicopathological tumor features or patient prognosis [23–26].

Despite conflicting data, research reveals that the identification of changes in cancer metabolism reprogramming based on GLUT isoform expression, as well as the oncogenic mechanisms leading to increased aerobic glycolysis, may be considered as potential new therapeutic targets for treatment strategies. They may also be used in synergy with conventional treatments to alter and/or inhibit cancer invasiveness and progression. Therefore, many authors propose the suppression of *SLC2A1* and *SLC2A3* genes by the use of antisense oligodeoxynucleotides which decrease glucose uptake, inhibit the proliferation of malignant cells, and enhance of radiosensitivity and chemosensitivity. This in turn allows the optimal treatment modality of neoplastic lesions, between them cancers of the head and neck region [12, 27, 28].

Less controversy surrounds the expression of HIF-1α, the endogenous regulator of glucose metabolism mechanisms in hypoxia conditions, with regard to clinicopathological parameters in various types of head and neck neoplasms. Previous studies indicate that cancer cells appear to display overexpression of this metabolic hypoxia-related protein in most tumor cases, and a high HIF-1α level is related to an enhanced grade of tumor aggressiveness, higher incidences of metastasis, and poor prognosis [26, 29–31]. However, a literature review reveals the conflicting results related to HIF-1α expression and its relationship with clinical features in various neoplastic lesions [4, 32, 33].

Unfortunately, it is hard to find literature data which clearly demonstrates the clinical importance of the relationship between HIF-1α level and GLUT isoform activity, as well as a precise multifactorial histological estimation of their influence on neoplastic aggressiveness in head and neck cancers. Hence, more studies are needed to elucidate the biological functions of both GLUTs and regulatory molecule HIF-1α in the carcinogenic process, and their possible clinical significance as parameters of tumor invasiveness and prognostic factors in this type of neoplasm.

The aim of this study was to determine the messenger RNA (mRNA) expression of *SLC2A1* and *SLC2A3* genes coding GLUT isoform 1 (GLUT1) and isoform 3 (GLUT3), as well as the regulatory HIF-1α gene and the levels of their corresponding proteins, to investigate their influence on tumor aggressiveness and patient prognosis in squamous cell laryngeal cancer.

## Materials and methods

### Patients and samples

The study material constituted 106 fresh biopsy tissue samples obtained from squamous cell laryngeal carcinoma (SCC) cases, and the control constituted 73 tissue samples from a morphologically estimated noncancerous laryngeal mucosa (NCM) from individuals who were qualified for total laryngectomy. According to ethical rules, the adjacent normal epithelium of the larynx in patients qualified for partial laryngectomy was not sampled. The patients (100 men and 6 women, mean age 62.4 ± 9.1 years) were recruited between January 2003 and December 2011 and were under treatment at the Department of Otolaryngology and Laryngological Oncology, Medical University of Łódź, Poland. All individuals had a confirmed diagnosis of laryngeal carcinoma based on histopathological evaluation and had undergone partial or total laryngectomy, depending on the extent of neoplastic lesions. All fresh samples were stored at −80 °C before the analyses. The tissue specimens collected in the operation room were prepared and evaluated by an experienced pathologist. Normal laryngeal tissues were collected from the sites as far as possible from the margins of the tumor by individually harvesting samples from presumptive noncancerous regions. Hematoxylin and eosin (H&E)-stained sections provided histological confirmation of noncancerous and cancerous tissues. The criteria for patient participation in this study were as follows: (1) a pathologically confirmed diagnosis of squamous cell planoepitheliale carcinoma; (2) primary surgical resection without receiving prior immunotheraphy, radiotheraphy, or chemotherapy; (3) absence of distant metastases and second primary neoplasms; (4) a negative history of previously diagnosed with other types of primary cancers; and (5) a negative history of recurrences of laryngeal cancer. Informed consent was obtained from each subject. The investigations were performed with the approval of the Bioethical Commission of the Medical University of Łódź and the National Science Council, Poland (approval No RNN/60/13/KE). In all cases, surveys were performed to complete the cancer registry database. The database catalog was queried every 2 months and identified all histopathologically confirmed incident primary squamous cell laryngeal cancer cases reported within 4 months of diagnosis preceding the recruitment. The sociodemographic features of the study subjects are shown in Table 1.

**Table 1:** Sociodemographic characteristics of patients with laryngeal carcinoma

Characteristic		Number of patients (%)
Sample size		106
Median age (range)	62.4 years (45–83)	
Median overall survival (range)	59.3 months (28–79)	
Gender	Male	100 (94.3)
	Female	6 (5.7)
Smoking status^a^	Current	78 (73.6)
	Former	23 (21.7)
	Never	5 (4.7)
Alcohol intake^b^	Never/rare	3 (2.8)
	Light	16 (15.1)
	Moderate	55 (51.9)
	Heavy	27 (25.5)
	Ex-drinkers	5 (4.7)
Surgical treatment	Total laryngectomy	73 (68.9)
	Partial laryngectomy	33 (31.1)
	Neck dissection (−)	60 (56.6)
	Neck dissection (+)	46 (43.4)
	Selective neck dissection	42 (39.6)
	Radical neck dissection	4 (13.8)
Survival	≥5 years	69 (86.8)
	<5 years	37 (13.2)
Local recurrences	No	93 (87.7)
	Yes	13 (12.3)
Nodal recurrences	No	94 (88.7)
	Yes	12 (11.3)

^a^Smoking was grouped into “current,” “former,” and “never” based on self-reported usage. Participants who reported smoking at least 100 cigarettes in their lifetime and who, at the time of survey, smoked either every day or some days were defined as current smoker. Participants who reported smoking at least 100 cigarettes in their lifetime and who had not been smoking for at least 3 months were defined as former smoker. Participants who reported never having smoked 100 cigarettes were defined as never smoker

^b^Never/rare, <1 unit/week; light, 1–8.9 units/week; moderate, 9–17.9 units/week; heavy, ≥18 units/week; where 1 unit = 22 g ethanol

### Histological classification and morphological features

Archival formalin-fixed paraffin-embedded tissue samples were used for the histological classification of tumors. All specimens were assessed according to the criteria conducted in accordance with the AJCC TNM classification of 2010 for laryngeal cancers [34]. Morphological estimation was performed on H&E-stained sections in the most invasive, peripheral zones of the tumor, according to tumor front grading (TFG), which is one of the most reliable pathological methods for the analysis of neoplastic progress and determination of the dynamics of tumor growth, as well as a reasonably precise prognostic factor in laryngeal carcinoma [35]. The histological evaluation considers the mode and depth of invasion as well as total TFG score. The factors were assessed in at least five different regions of the peripheral part of the tumor (magnification ×200, number of mitoses magnification ×400). Each factor was graded according to a scale ranging from 1 to 4. The numeric morphological TFG score was computed as the sum of tumor-related features (cytoplasmic differentiation, nuclear polymorphism, number of mitoses) and adjacent stroma-related characteristics of the peripheral edge of tumor infiltration (mode of invasion, depth of invasion, and plasmalymphocytic infiltration), with a maximum score of 24 points. According to the TFG total score, tumors were divided into four groups: 6–9, 10–13, 14–17, 18–21, and >22 TFG points. The clinicopathological characteristics of the laryngeal cancers are shown in Table 2.

**Table 2:** Clinicopathological characteristics of laryngeal cancers

Characteristics		Cases (%)
Tumor size (pT status)	pT1	10 (9.4)
	pT2	29 (27.4)
	pT3	33 (31.1)
	pT4	34 (32.1)
Lymph node metastases (pN status)	pN0	84 (79.2)
	pN1-3	22 (20.8)
Degree of differentiation (grade)	G1	14 (13.2)
	G2	82 (77.4)
	G3	10 (9.4)
TFG total score^a^	6–9 points	5 (4.7)
	10–13 points	39 (36.8)
	14–17 points	39 (36.8)
	18–21 points	23 (21.7)
	>22 points	0
Mode of invasion	1—Well-defined borderline	14 (13.2)
	2—Less marked borderline	34 (32.1)
	3—No distinct borderline	37 (34.9)
	4—Diffuse growth	21 (19.8)
Depth of invasion	1—Carcinoma in situ	5 (4.7)
	2—Microinvasion	35 (33.0)
	3—Nodular into submucosa	27 (25.5)
	4—Deep invasion (cartilage infiltratio)	39 (36.8)

^a^The numeric morphological TFG score was computed as the sum of six tumor-related features (cytoplasmic differentiation, nuclear polymorphism, number of mitoses) and adjacent stroma-related characteristics of the peripheral edge of tumor infiltration (mode of invasion, depth of invasion, and plasmalymphocytic infiltration). The factors of TFG were assessed in at least five different regions of the peripheral part of the tumor (magnification ×200, number of mitoses magnification ×400). Each factor was graded according to a scale ranging from 1 to 4

### RNA isolation and cDNA synthesis

The tissue specimens collected in the operation room were prepared and evaluated by an experienced pathologist. Samples were stored at −80 °C until RNA preparation. Total RNA was isolated using Trizol® Reagent (Sigma-Aldrich, USA) according to manufacturer’s protocol and quantified spectrophotometrically. RNA was eluted in 20 μl RNase-free water, quantified by spectrophotometry at 260 nm and stored at −20 °C. RNA with a 260/280 nm ratio in the range 1.8–2.0 was considered high quality. First-strand complementary DNAs (cDNAs) were obtained by reverse transcription of 1 μg of total RNA using RevertAid™ first-strand cDNA synthesis kit (Fermentas International, Lithuania) following the manufacturer’s protocol.

### Quantitative real-time RT-PCR

Real-time gene expression analysis of target genes (*SLC2A1*, *SLC2A3*, and *HIF-1α*) was performed using TaqMan® Gene Expression Assays (Applied Biosystems, USA) according to manufacturer’s instructions. The hypoxanthine phosphoribosyltransferase 1 (*HPRT1*) gene was used as internal control. The assay numbers for these genes were as follows: Hs00892681_m1, Hs00359840_m1, Hs00936368_m1, and Hs02800695_m1. Each PCR reaction was performed in a 10-μl volume that included 5 μl of 2× TaqMan Universal PCR MasterMix (Applied Biosystems, USA), 4.5 μl of water diluted cDNA template (50 ng), and 0.5 μl of TaqMan® Gene Expression Assay consisted of a pair of unlabeled PCR primers and TaqMan probe with a FAM™. The RT-qPCR reaction was carried out using the Mastercycler ep realplex (Eppendorf) under the following conditions: denaturation for 10 min at 95 °C followed by 50 cycles of 15 s at 95 °C, 1 min annealing and extension at 60 °C. Relative RNA quantification was performed using the ΔCt method. ΔCt (Ct_gene_ − Ct_*HPRT1*_) values were recalculated into relative copy number values (number of *SLC2A1*, *SLC2A3*, and *HIF-1α* mRNA copies per 1000 copies of *HPRT1* mRNA).

### *SLC2A1* and *SLC2A3* gene copy number quantification

To determine the *SLC2A1* and *SLC2A3* gene amplification, copy number quantification was carried out using quantitative real-time PCR Mastercycler ep realplex (Eppendorf) with the glucokinase (*GCK*) gene used as the reference gene. The real-time PCR primers are as follows: *SLC2A1* (f) 5′-TGTGCAACCCATGAGCTAA-3′, *SLC2A1* (r) 5′-CCTGGTCTCATCTGGATTCT-3′; *SLC2A3* (f) 5′-TTCGTCTCTAGCCTGCACTG-3′, *SLC2A3* (r) 5′-ACACAACTTCTCCGGGTGAC-3′; *GCK*(f) 5′-CGGATGCAGAAGGAGATGGA-3′, and *GCK*(r) 5′-CATCTTCACACTGGCCTCTTCA-3′. Real-time PCR was performed in 50-μl reaction volumes that contained 2× Power SYBR Green PCR Master Mix (Applied Biosystems, USA) and 0.9 mM forward and reverse primers. PCR conditions were as follows: 5 s at 95 °C followed by 40 cycles consisting of 15 s at 95 °C and 30 s at 60 °C. ΔCt was calculated by a Ct value of GCK taking away that of *SLC2A1* and *SLC2A3* and three or more ΔCt was defined as amplified.

### Western blotting analysis

The protein content of the tissue homogenate fraction was estimated by means of the modified Lowry procedure [36] using bovine serum albumin (BSA) as standard. The samples (50 μg protein/lane) of homogenates were resolved by 8 % SDS-PAGE and electroblotted onto Immobilon-P transfer membranes (Millipore, Bedford, MA, USA). The blots were incubated 1 h with rabbit polyclonal anti-GLUT1 (Abcam, UK), mouse monoclonal anti-GLUT3 (Santa Cruz Biotechnology, Inc., USA) or rabbit polyclonal anti-HIF-1α antibodies (Santa Cruz Biotechnology, Inc., USA) in a 1:1000, 1:400, and 1:1000 dilution, respectively. After being washed three times with Tris-buffered saline with Tween-20 (TBST), the membranes were incubated 1 h with goat anti-rabbit or anti-mouse antibodies conjugated with horseradish peroxidase (1:5000 dilution). The membranes were again washed three times with TBST and incubated with peroxidase substrate solution (3,3′-diaminobenzidine (DAB)). Gel-Pro® Analyzer software (Media Cybernetics Inc., USA) was used for densitometry analysis of protein bands. The integrated optical density (IOD) of the bands, in a digitized picture, was measured. For the immunoblot analysis, an IOD less than 5 was taken as negative.

All RT-PCR and Western blot reactions were repeated three times for each sample.

### Statistical analysis

The statistical analyses were performed using STATISTICA version 9.0 (StatSoft, Poland). Since levels of expression in endometrial and breast cancer specimens did not follow a normal distribution (Kolmogorov-Smirnov test), nonparametrical statistical tests were applied (Mann-Whitney *U* test, Spearman’s rank analysis). Kruskal-Wallis test with post hoc multiple comparisons was used according to clinical data. Kaplan-Meier survival analysis was performed to determine the association of *SLC2A1*, *SLC2A3*, and *HIF-1α* mRNA expression with overall survival. The cutoff value was established to be the median of *SLC2A1*, *SLC2A3*, and *HIF-1α* mRNA level. The survival curves were compared between two groups: high (≥ median value) and low (< median value) expression using log-rank tests. Distribution of quantitative variables was described using means and standard deviations. A *p* value <0.05 was considered as statistically significant.

## Results

### *SLC2A1*, *SLC2A3*, and *HIF-1α* gene expression in neoplastic and noncancerous tissues

The mRNA expression of genes *SLC2A1*, *SLC2A3*, and *HIF-1α* in either SCC or NCM was estimated by real-time quantitative PCR analysis with *HPRT1* gene applied as a reference. A positive expression of *SLC2A1* and *SLC2A3* transcripts was confirmed in 83.9 % (89/106) and 82.1 % (87/106) samples of laryngeal cancer, respectively. In the case of normal tissue, positive expression of GLUT1 and GLUT3 mRNA was demonstrated in 49.3 % (36/73) and 43.8 % (32/73) of samples, respectively. Positive expression of *HIF-1α* gene was noted in 71.7 % (76/106) of SCC and in 9.6 % (7/73) of NCM, respectively. Thus, positive expression of both GLUT transcripts and HIF-1α was more frequent in SCC than in NCM.

**Fig. 1:**
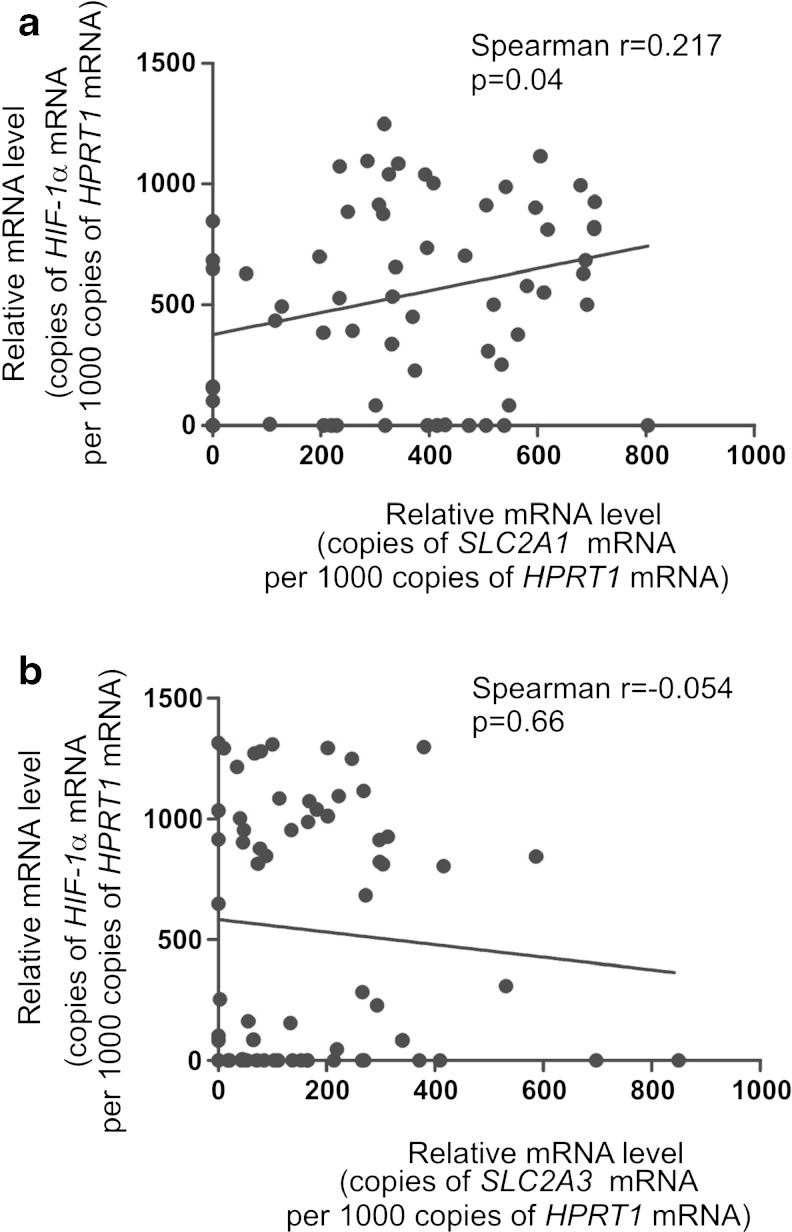
Spearman’s rank analysis results for *HIF-1α* mRNA expression level in relation to *SLC2A1* (**a**) and *SLC2A3* (**b**) genes in laryngeal cancer

A significant difference was noted in the levels of GLUT1 and GLUT3 mRNA in laryngeal cancer tissue compared to adjacent normal laryngeal tissue (*p* < 0.001 and *p* < 0.001, for the *SLC2A1* and *SLC2A3* genes, respectively). The relative expression of *SLC2A3* transcripts was much lower than *SLC2A1* in both SCC and NCM. Similarly, mean mRNA expression of the *HIF-1α* gene in neoplastic tissue was also higher than that in normal laryngeal tissue, but the difference was not significant (*p* > 0.05). Mean GLUT1, GLUT3, and *HIF-1α* gene expression in SCC and NCM, as well as the results of statistical analysis, are summarized in Table 3. No significant correlation was found between GLUT1 and GLUT3 mRNA expression levels (Spearman’s rank analysis, *p* > 0.05). However, a significant correlation was found between expression of the *HIF-1α* gene with *SLC2A1* transcripts (Spearman’s rank analysis, *r* = 0.21, *p* = 0.04), but not *SLC2A3* transcripts (Spearman’s rank analysis, *p* > 0.05), as determined by real-time PCR. The expression of *HIF-1α* mRNA in relation to *SLC2A1* and *SLC2A3* genes in laryngeal cancer is shown in Fig. 1.

**Table 3:** Mean *SLC2A1*, *SLC2A3*, *HIF-1α* gene and related protein expression in cancerous and normal tissues

	mRNA gene expression (copies of gene mRNA per 1000 copies of *HPRT1* mRNA)	*p* value	Protein expression (integrated optical density)	*p* value
Gene/Protein	*SLC2A1*		GLUT1	
SCC	353.72 ± 226.42		97.83 ± 35.51	
NCM	156.42 ± 92.15	<0.001	48.17 ± 32.67	0.032
	*SLC2A3*		GLUT3	
SCC	176.09 ± 173.38		118.63 ± 64.22	
NCM	21.56 ± 11.21	<0.001	0	−
	*HIF-1α*		HIF-1α	
SCC	564.68 ± 481.99		179.1 ± 245.34	
NCM	44.29 ± 153.69	<0.0001	76.21 ± 97.44	0.11

Results are given as mean ± standard deviation

*SCC* squamous cell laryngeal cancer, *NCM* noncancerous laryngeal mucosa

Gene copy number analysis revealed that *SLC2A1* gene amplification was observed in 2 % (2/106) of laryngeal cancer cases, but in none of the normal tissue samples. In the case of *SLC2A3* gene, amplification was confirmed neither in cancerous nor normal tissues.

### Association between *SLC2A1*, *SLC2A3*, and *HIF-1α* transcripts and tumor behavior

The expression of *SLC2A1*, *SLC2A3*, and *HIF-1α* transcripts with regard to relevant clinicopathological parameters in laryngeal cancers is shown in Table 4. The mRNA level of genes coding GLUT1 and GLUT3 significantly increased together with the degree of histological differentiation (*p* = 0.0005 and *p* = 0.003, respectively). Significant differences were noted between grade 1 and grade 3 tumors as well as between grade 1 and grade 2 positive cancers with regard to *SLC2A1* expression (*p* < 0.001 and *p* < 0.05, respectively), as well as between grade 3 and both grade 1 and 2 positive cancers with regard to *SLC2A3* expression (*p* < 0.01 and *p* < 0.05, respectively). No differences in *SLC2A1* and *SLC2A3* gene level were noted with respect to other pTNM parameters (*p* > 0.05). However, the results showed that more advanced tumors (pT3-4, pN1-3) were more likely to be more frequently positive and to have a higher mean levels for both *SLC2A1* and *SLC2A3* transcripts than less advanced cancers (pT1-2, N0).

**Table 4:** Expression of *SLC2A1*, *SLC2A3*, and *HIF-1α* gene in laryngeal cancers

Characteristics		*SLC2A1*	*SLC2A3*	*HIF-1α*
		Positive/total (%)	Positive/total (%)	Positive/total (%)
Tumor size (pT status)	pT1	8/10 (80.0)	8/10 (80.0)	2/10 (20.0)
	pT2	24/29 (82.8)	23/29 (79.3)	13/29 (44.8)
	pT3	28/33 (84.8)	28/33 (84.8)	27/33 (81.8)
	pT4	29/34 (85.3)	28/34 (82.4)	34/34 (100.0)
Lymph node metastases (pN status)	pN0	68/84 (80.9)	73/84 (86.9)	54/84 (64.3)
	pN1-3	21/22 (95.5)	14/22 (63.6)	22/22 (100.0)
Degree of differentiation (grade)	G1	10/14 (71.4)	11/14 (78.6)	6/14 (42.8)
	G2	69/82 (84.1)	67/82 (81.7)	62/82 (75.6)
	G3	10/10 (100.0)	9/10 (90.0)	8/10 (80.0)
TFG total score	<14 points	35/44 (79.5)	29/44 (65.9)	17/44 (38.6)
	≥14 points	54/62 (87.1)	58/62 (93.5)	59/62 (95.2)
Mode of invasion	1–2 points	32/48 (66.7)	35/48 (72.9)	18/48 (37.5)
	3–4 points	57/58 (98.2)	52/58 (89.6)	58/58 (100.0)
Depth of invasion	1–2 points	33/40 (82.5)	26/40 (65.0)	14/40 (35.0)
	3–4 points	56/66 (84.8)	61/66 (92.4)	62/66 (93.9)
Survival	≥5 years	58/69 (84.1)	64/69 (92.8)	40/69 (58.0)
	<5 years	31/37 (83.8)	30/37 (81.1)	36/37 (97.3)
Local recurrences	No	79/93 (84.9)	76/93 (81.7)	63/93 (67.7)
	Yes	12/13 (92.3)	11/13 (84.6)	13/13 (100.0)
Nodal recurrences	No	76/94 (80.9)	76/94 (80.9)	64/94 (68.1)
	Yes	11/12 (91.7)	11/12 (91.7)	12/12 (100.0)

**Fig. 2:**
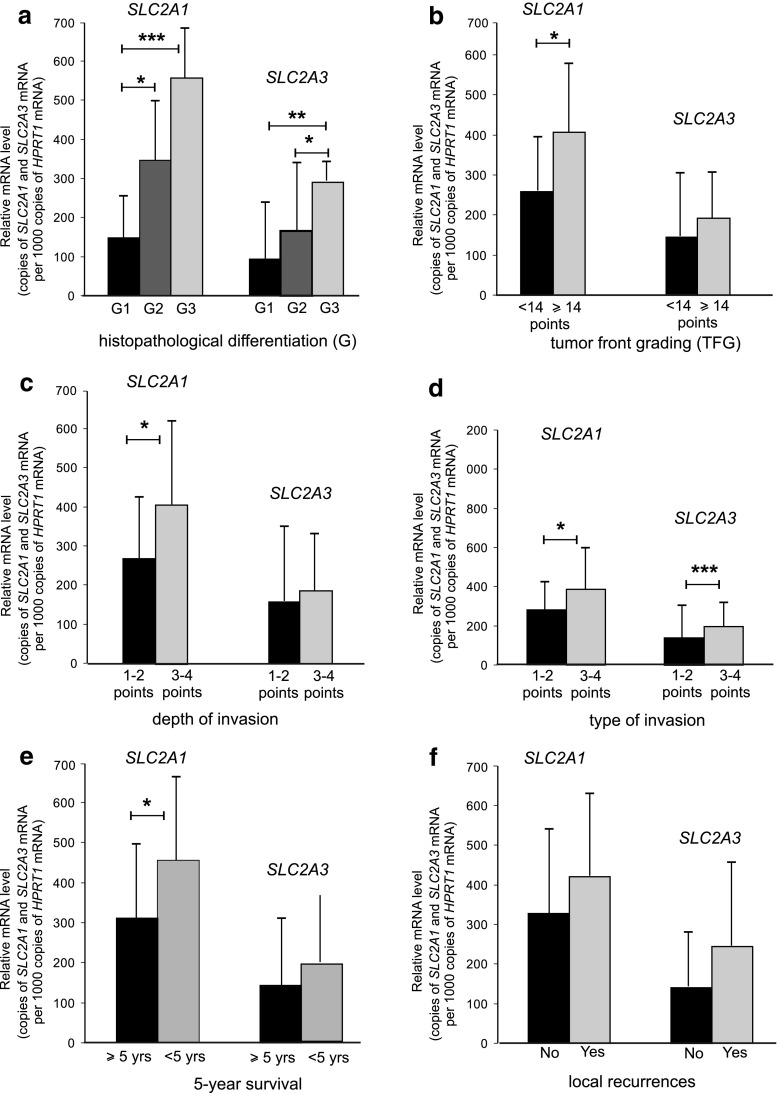
Expression of *SLC2A1* and *SLC2A3* mean mRNA measured by real-time PCR in laryngeal cancers; a comparison between subgroups with histological differentiation (grade) (**a**), total score of tumor front grading (**b**), depth of invasion (**c**), mode of invasion (**d**), 5-year survival (**e**), and local recurrences (**f**). *Graphs* represent mean ± standard deviation. *p < 0.005, **p < 0.001, ***p < 0.0001

*SLC2A1* mRNA expression was significantly higher in laryngeal TFG cancers with a total score ≥14 points than less aggressive ones (*p* = 0.021). Moreover, SCC characterized by deep invasion with submucosa or cartilage infiltration (3–4 points) and by diffuse growth or invasion with no distinct borderlines (3–4 points) demonstrated significantly higher levels *SLC2A1* mRNA in comparison with less invasive tumors (*p* = 0.028 and *p* = 0.021, respectively). Mean *SLC2A3* gene expression was significantly higher in more disseminated invasion type cancers (*p* = 0.0001). Furthermore, *SLC2A1* transcript level was also significantly related to disease-free survival when compared <5- and ≥5-year categories. Increased GLUT1 expression was found to be associated with a survival time of less than 5 years (*p* = 0.012). *SLC2A1* expression was found to have no connection with the incidence of local and nodal recurrences (*p* > 0.05). No significant differences were found between *SLC2A3* mRNA level and either recurrence or 5-year disease-free survival (*p* > 0.05). The association between clinicopathological parameters and the expression of *SLC2A1* and *SLC2A3* transcripts, as well as with the results of the statistical analysis, are shown in Fig. 2.

**Fig. 3:**
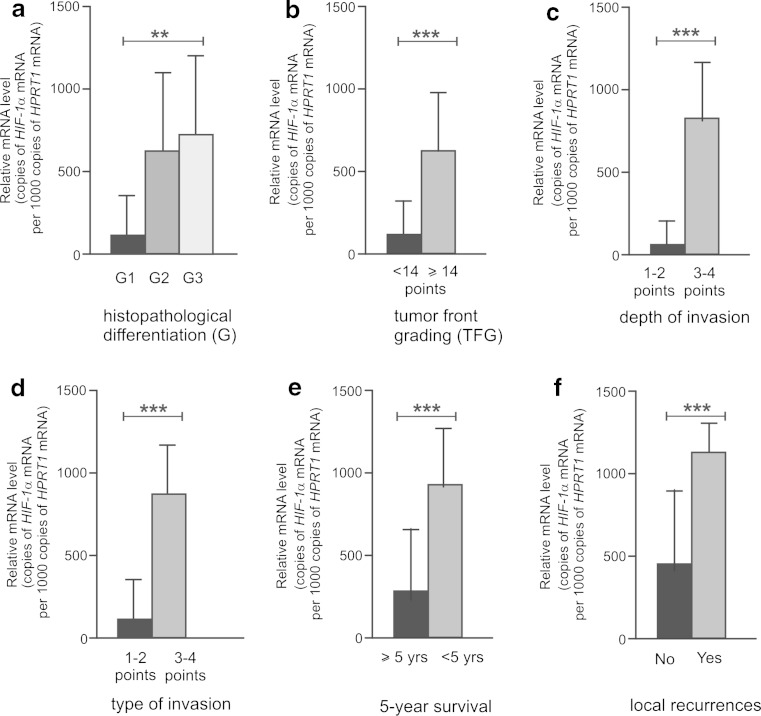
Expression of *HIF-1α* mean mRNA measured by real-time PCR in laryngeal cancers; a comparison between subgroups with histological differentiation (grade) (**a**), total score of tumor front grading (**b**), depth of invasion (**c**), mode of invasion (**d**), 5-year survival (**e**), and local recurrences (**f**). Graphs represent mean ± standard deviation. *p < 0.05, **p < 0.01, ***p < 0.001

The *HIF-1α* mRNA level significantly increased with the size of the primary tumor (pT3-4) (*p* < 0.001), the presence of nodal metastases (pN1-3) (*p* < 0.001), and tumor grade (*p* = 0.004). Moreover, laryngeal cancers with TFG total score ≥14 points, deep invasion (3–4 points), and disseminated growth (3–4 points) were noted to have significantly higher *HIF-1α* gene level compared to less invasive tumors (*p* < 0.001, *p* < 0.001, and *p* < 0.001, respectively). In addition, increased expression of the gene coding HIF-1α was found to promote a survival time of less than 5 years (*p* < 0.001) and the presence of local and nodal recurrences (*p* < 0.001 and *p* < 0.001, respectively). The relationship between various clinicopathological parameters and *HIF-1α* gene expression, as well as with the results of the statistical analysis, is shown in Fig. 3.

**Fig. 4:**
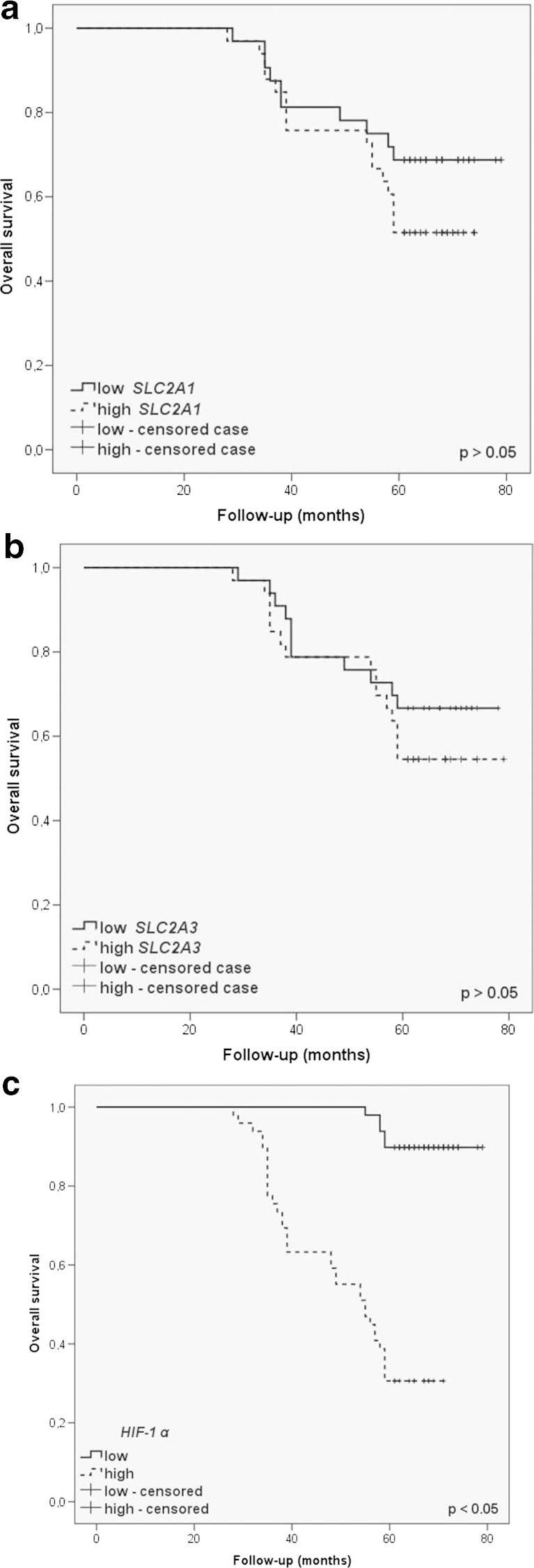
Kaplan-Meier plots of overall survival for categorized by *SLC2A1* (**a**), *SLC2A3* (**b**), and *HIF-1α* (**c**) mRNA expression in homogenate samples of laryngeal cancer tissues. The cutoff value was established to be the median of the *SLC2A1*, *SLC2A3*, and *HIF-1α* mRNA levels. The survival curves were compared between two groups: high (≥ median value) and low (< median value) expression

Moreover, overall survival was analyzed through the Kaplan-Meier plots, as shown in Fig. 4. Mean overall survival time was 67.8 and 60.6 months in low and high *SLC2A1* expression groups, respectively. Similarly, mean overall survival time was 66.4 months in low *SLC2A3* mRNA expression and 64.2 months in high *SLC2A3* expression. No significant differences were found in GLUT1 and GLUT3 expression in relation to prognosis (*p* > 0.05). However, the results revealed a trend of worse overall survival rate in cases with a high expression of the GLUT1 and GLUT3. Mean overall survival time was 76.8 months in low *HIF-1α* mRNA expression and 52.5 months in high *HIF-1α* expression. A significant shorter overall survival for a higher *HIF-1α* gene expression was estimated (*p* < 0.001).

### GLUT1, GLUT3, and HIF-1α protein expression in neoplastic and noncancerous tissues

**Fig. 5:**
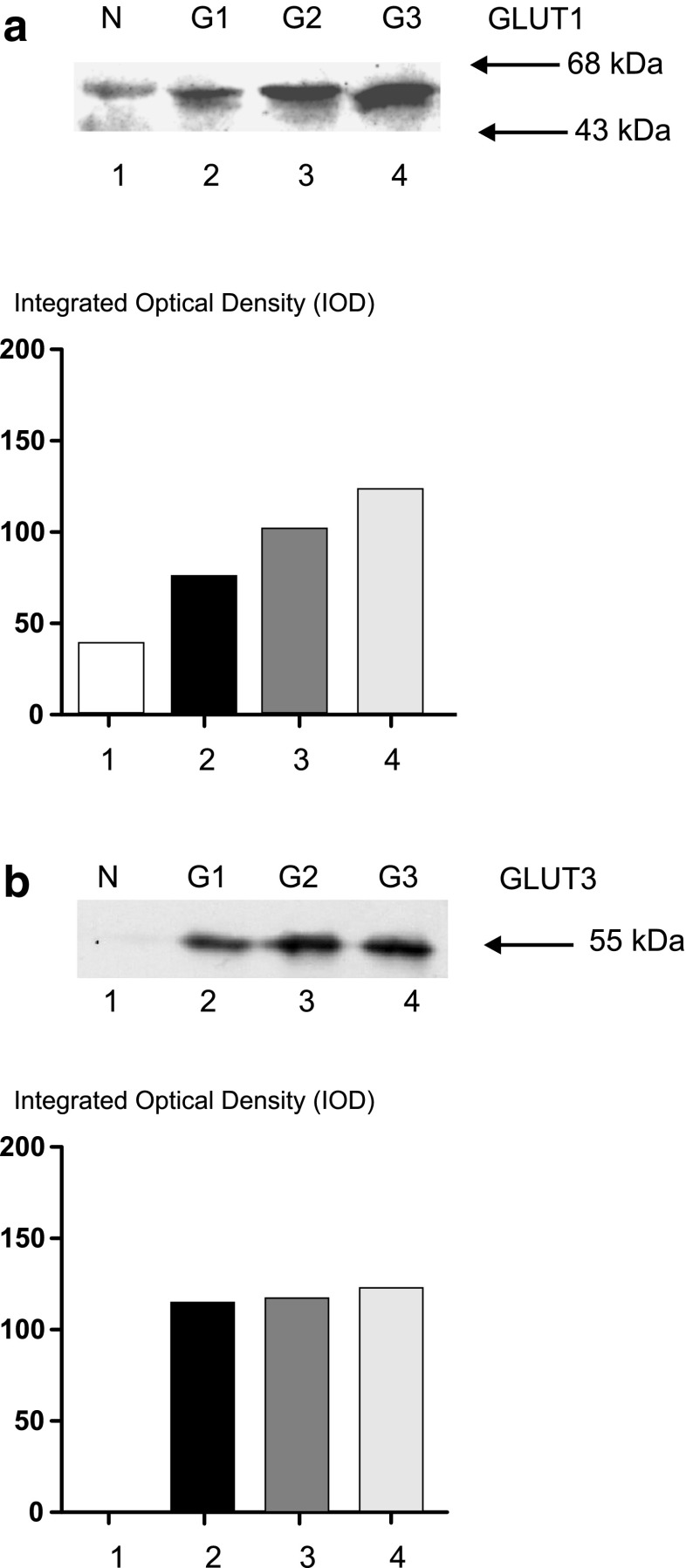
Representative immunoblot and quantitative analysis results of GLUT1 (**a**) and GLUT3 (**b**) protein expression in homogenate samples (50 μg protein loaded per lane) of normal (*lane 1*) and laryngeal cancer tissues (*lanes 2–4*) in relation to tumor grade. *Lower panels* show the results of quantitative densitometric analysis: *lane 1*, normal laryngeal mucosa, *lanes 2–4*, laryngeal cancer samples classified according to the three-grade morphological system as G1 (well-differentiated tumor), G2 (moderately-differentiated tumor), and G3 (poorly-differentiated tumor), respectively

In the next stage, expression of GLUT1, GLUT3, and HIF-1α was examined on the protein level using Western blotting analysis, in homogenate samples of both SCC and NCM specimens. The 32 tissue samples selected for quantitative densitometric analysis were representative for whole group studied and characterized by similar distribution, based on the pTNM classification. Of the carcinoma tissue samples, 34.4 % (11/32) and 59.4 % (19/32) showed GLUT1- and GLUT3-positive expression, respectively. In the case of matched normal laryngeal tissue, GLUT1 protein expression was observed in 31.3 % of samples and GLUT3 in none. The results of the quantitative densitometric analysis of the intensity of GLUT1 and GLUT3 bands in homogenates of normal and cancerous tissues are shown in Fig. 5. Positive HIF-1α protein expression was noted in 62.5 % (20/32) of SCC and in 15.6 % of NCM, respectively. Mean protein expression of GLUT1 and HIF-1α in SCC were higher compared to NCM, but only GLUT1 protein level was found to be significantly different (*p* = 0.032). Mean GLUT1, GLUT3, and HIF-1α protein expression in SCC and NCM and statistical analysis results are shown in Table 3.

### Association between GLUT1, GLUT3, and HIF-1α proteins and the tumor behavior

GLUT and HIF-1α expression was also compared with the pTNM classification of laryngeal cancer cases, since the tumor stage is currently the only accepted prognostic marker. Despite the relatively small size of positive results for the presence of proteins studied and the small proportion of subcategories of pTNM classification, the results show a clear relationship between quantitative densitometric results and stage parameters.

The results reveal a trend of more advanced tumors to have more common positive expression of GLUT1, GLUT3, and HIF-1α proteins, according to Western blot analysis. The positive expression of GLUT1 and GLUT3 proteins in SCC samples according to pTNM categories was as follows: 33.3 % (5/15) in pT1-T2 and 35.3 % (6/17) in pT3-T4 tumors, 18.8 % (4/22) in pN0 and 70 % (7/10) in pN1-3 for GLUT1, and 46.7 % (7/15) in pT1-T2 and 70.6 % (12/17) in pT3-T4 tumors, 50 % (11/22) in pN0 and 80 % (8/10) in pN1-3 for GLUT3. It should be also emphasized that higher graded tumors were found to demonstrate higher expression of the GLUT1 protein, but not GLUT3. The results concerning GLUT1 and GLUT3 expression in homogenate samples of laryngeal cancer tissues in relation to tumor grade are shown in Fig. 5.

Similar trends were demonstrated for the HIF-1α protein. The levels of positive HIF-1α expression according to pTNM categories and histological grade was as follows: 26.7 % (4/15) in pT1-T2 and 94.1 % (16/17) in pT3-T4 tumors, 54.5 % (12/22) in pN0 and 80 % (8/10) in pN1-3 and 10 % (1/10) for well-differentiated tumors, 75 % (9/12) for moderately-differentiated cancers, and 100 % (10/10) for poorly-differentiated tumors.

## Discussion

In the last decade, a growing body of evidence indicates that increased level of glycolytic activity observed under aerobic conditions (the Warburg effect) needed to meet the energy requirements of tumor cells and the endogenous markers of metabolism/hypoxia may contribute to the development, proliferation, angiogenesis, and progression of various types of head and neck cancers [1–5]. Studies have linked GLUT function to cancer cell dynamics and tumor pathology, yet findings remain limited and often divergent, especially regarding the impact of their specific isoforms on tumor invasion and prediction of patient outcome [15–26]. The activity of the glycolytic pathway may also be responsible for radioresistance and chemoresistance and thus may affect patient prognosis [27, 28, 37].

Our study documents for the first time the relationship between the expression of GLUT1 and GLUT3 isoforms in the fresh human biopsy material and the dynamics of tumor growth in laryngeal cancer, according to a precise, multifactorial histological analysis of tumor front aggressiveness. Importantly, the material studied constitutes a homogeneous and numerous groups of head and neck cancers sharing the same origin. This composition of the study groups adds to the value of this research, when the difficulties in obtaining fresh human biopsy tissues such as cancerous and matched normal tissues are taken into consideration. It should be also noted that, due to these difficulties, the overwhelming majority of previous studies have used cell cultures and paraffin-embedded archival material, often also limited in number, which doubtlessly have an impact on the final results. In this study, the protein expression in less material due to this limitation complements research on the genes studied.

A literature search indicates that no studies on the amplification of the *SLC2A1* and *SLC2A3* genes in fresh tissues from the head and neck region have yet been performed. Since copies of the studied genes were found in only 2 % cases of laryngeal carcinoma, it can be concluded that *SLC2A1* and *SLC2A3* amplification does not affect the expression of GLUT1 and GLUT3 in this type of neoplasm.

The resulting data concerning expression of GLUT isoforms in laryngeal cancer resembles the findings of other researchers [28, 38–40]. However, it should be noted that a literature survey revealed only a few studies on these relationships in head and neck cancer. For instance, Zhou et al. [22] reported that the expression of the GLUT1 and GLUT3 genes were significantly higher in cancerous tissue than in adjacent normal tissue in 38 head and neck cancer samples. A similar result was reported by Burstein et al. [38] for 27 cases of squamous intraepithelial neoplasia and 26 invasive carcinomas of the head and neck region. In this study, upregulation of GLUT1 at the protein level was established in tumor specimens compared with normal epithelium, characterized by negativity or weak/equivocal staining. Previous studies have also reported a lower prevalence or absence of HIF-1α in noncancerous tissue of various head and neck tumors [39, 40]. For example, Zhang et al. [39] observed an overexpression of both GLUT1 and HIF-1α proteins in 85 neoplastic tissues of oral squamous cell cancer and conclude these metabolism/hypoxia-related proteins can be predictive markers for malignant conversion of oral premalignant epithelial dysplasia. Similarly, Xie et al. [40] confirmed enhanced immunostaining for HIF-1α in 56 laryngeal cancer tissues and its negative expression in normal mucosa specimens.

In addition, a literature review reveals a wide range of findings concerning expression rate and distribution of GLUTs in various head and neck cancers. The data concerning the expression of GLUT1 and GLUT3 transcripts or, most often, protein immunoexpression identified in laryngeal cancer cases were found to be very similar to ours: the mean frequency of GLUT1 was in the range 50.3–98 % while GLUT3 positive expression was less so [8, 18, 21, 22, 24–26]. The reasons for lower GLUT3 mRNA/protein expression in various cancer specimens may be, on the one hand, the activity of other GLUT isoforms, i.e., GLUT1, which are responsible for the glucose uptake and glycolytic metabolism, and on the other hand, due to heterogeneity of GLUT expression within tumor tissues. In addition, laryngeal cancers often have numerous foci of necrosis, where glucose metabolism is less pronounced and may result in lower expression of the less active GLUT3 isoform [41]. Therefore, our analysis has been restricted to areas without tumor necrosis. It is not without significance that HIF-1α activity, which determines GLUT expression, is also not ubiquitous and heterogenous in cancerous tissue and increases with the distance of the sampling site from the blood vessels [42].

Studies have confirmed the role of GLUT isoforms, mainly GLUT1, in head and neck neoplastic lesions and both cancer development, tumor progression and patient prognosis, but many demonstrate equivocal and divergent results [15–26]. Importantly, most which address invasive behavior concern only standard clinical parameters (TNM classification, grade) and survival time.

Most studies confirm the increased expression of GLUTs in more advanced head and neck cancers, where a positive association was found for clinical variables including tumor grade and stage [8, 18, 22, 26, 43]. For instance, Wu et al. [8] reported enhanced GLUT1 isoform expression in a studied population of 49 laryngeal cancer cases and identify a positive association between GLUT1 immunostaining and primary tumor site, lymph node invasion, and recurrences. Similarly, Ayala et al. [18] estimated a positive association of GLUT3 immunoexpression with advanced clinical tumor stage and vascular embolization in laryngeal cancer. Kondo et al. [26] also revealed a correlation between increased GLUT1 IHC expression in primary oral cancer tissues with advanced nodal metastatic stage but, as identified by the present study, not with the survival rate. Zhou et al. [22] indicated that higher GLUT1 and GLUT3 mRNA levels in head and neck carcinomas may also have an effect on advanced clinical stage and positive lymph node status. The distributions of hypoxia/metabolic markers in oral tongue cancer described by Roh et al. [43] confirm those given in the present study. The researchers suggest that GLUT1 expression contributes to tumor thickness and nodal classification. Also, Demasi et al. [19] revealed that overexpression of GLUT1 corresponds to lower histological tumor differentiation in patients with salivary gland carcinoma, which confirms our results. Ciampi et al. [21] performed an analysis of GLUT expression in human thyroid primary carcinoma tissues and cell lines to determine the impact of GLUT1 on tumor histological differentiation. The authors note that the occurrence of positive GLUT1 expression is frequently characteristic of anaplastic tumors compared with well-differentiated cancers. By contrast, overexpression of GLUT3 mRNA in this cell cancer model corresponds to lower tumor grade. It was also determined that the protein level of this isoform in fresh tissue samples is not detectable. However, Ohba et al. [20] suggest that upregulation of GLUT1 protein level may play a crucial role in determining the depth of invasion but bears no relation with primary tumor size and nodal metastases in oral carcinoma.

Nevertheless, contrary results for GLUT1 and GLUT3 immunoexpression related to such clinical parameters as primary tumor size, extrathyroidal extension, and lymph node metastases has also been reported by Kaida et al. [23]. This data is confirmed by Kwon et al. [37] in a study of 42 early-stage laryngeal cancers, which notes that GLUT1 was not found to exert any influence on residual tumor or survival after radiotherapy. Subsequently, no association between GLUT1 level in malignant oesophageal cancer samples and pN stage was shown by Kobayashi et al. [16]. However, similar to our results, the authors reported enhanced GLUT1 immunostaining in individuals with more locally advanced carcinomas in this region of the head and neck.

Unfortunately, the relationship between GLUT level and prognosis was also found to be divergent in the head and neck cancer populations. The obtained data confirms that GLUT isoform expression was unrelated to mortality. The prognosis results described by Müssig et al. [25] were almost the same as those given in the present study. Higher GLUT1 immunoexpression has been found not to be associated with clinical outcome in the thyroid cancer population. Similarly, Schrijvers et al. [44] reported different findings regarding GLUT1 immunostaining in 91 early-stage glottic carcinomas treated with radiotherapy only. In this case, GLUT1 overexpression was found to be not significantly related to the clinical outcome parameters.

However, other researchers report different findings and conclude that GLUT activity may be implicated in patient survival time. For instance, Ohba et al. [20] suggested that upregulation of GLUT1 protein level may affect prognosis in patients with oral carcinoma. Similarly, Zhou et al. [22] observed that individuals having GLUT1 positive gene expression demonstrated considerably shorter overall survival, but no association was found between GLUT3 transcript and prognosis in the studies head and neck cancer populations.

Less controversy and fewer conflicting results concern the clinical and prognostic value of HIF-1α and its relationship with glucose metabolism molecules in head and neck cancers [8, 45]. Our study shows a strong positive correlation between the activity of HIF-1α and GLUT1, but not GLUT3, and indicates its association with a higher advancement of neoplastic lesions and poor patient outcome. The literature also offers a considerable body of evidence for the role of HIF-1α in carcinogenesis and the metabolic regulation of GLUTs, thus confirming our observations. For example, Wu et al. [8] demonstrated a significant correlation between HIF-1α and GLUT1 expression and report that increased hypoxia/metabolism markers are independent predictors of recurrences, lymph node metastases, and shortened survival in laryngeal cancer. Similarly, Yamada et al. [45] confirmed the significant coexpression of HIF-1α and GLUT1 in early-stage tumors in squamous cell oral cancers.

A few studies concerning HIF-1α expression in relation to tumor behavior and prognosis in head and neck carcinomas also confirm our findings [29–31]. Li et al. [31] reported that HIF-1α could be regarded as a potential predictor of a higher clinical stage and nodal metastases of laryngeal carcinoma. Also, Koperek et al. [30] suggested that HIF-1α contributes to a higher pN status and peritumoral/extrathyroidal infiltration, as well as angioinvasion in thyroid carcinoma. Ping et al. [29] noted that HIF-1α overexpression was found to be significantly related to stage, nodal metastases, depth of invasion, local recurrences, and clinical outcome in esophageal cancer.

Unfortunately, a few studies present inconsistent data regarding the status of HIF-1α as a biomarker of tumor invasiveness and prognosis [32, 33]. For example, Douglas et al. [32] noted a lack of prognostic effect of HIF-1α overexpression in patients with early squamous cell carcinoma of the glottis treated with radiotherapy. Also, Cabanillas et al. [33] observed that HIF-1α expression correlated with the tumor local extension but was not associated with tumor stage or lymph node metastases in supraglottic laryngeal cancer.

Finally, it should be also stressed that there are limitations to our study. While GLUT1 and GLUT3 expression could be considered as useful potential biomarkers for tumor behavior in laryngeal cancer, discrepancies exist for other tumors of this region due to variation of tumor types, histological differentiation status, and proliferative index, which cause differences in their biology. Admittedly, the results indicate the issue role of GLUTs and interactions with HIF-1α as their key regulator in cancers, but it is not clearly understood and there is a need for future research to clarify the relevance of GLUT proteins in carcinogenicity and the behavior of various tumors. Also, because of the limited amount of biological material in our study, GLUTs and HIF-1α were compiled with regard to protein expression only, with pTNM classification, the currently accepted prognostic parameters, being used in a smaller number of laryngeal cancer cases.

## Conclusions

In conclusion, our findings suggest that both the *SLC2A1* and *SLC2A3* genes, as well as the related GLUT1 and GLUT3 proteins, can affect the behavior of laryngeal cancer. Moreover, our findings indicate the importance of the expression of GLUTs as a significant factor in determining tumor aggressiveness, which should be taken into consideration in choosing alternative and optimal treatment modalities, i.e., extension of primary surgical procedure, and can contribute to prognosis in patients with cancer of the larynx. However, additional data on *SLC2A1* and *SLC2A3* genes and their products in head and neck carcinomas is needed to elucidate the biological function of GLUTs in carcinogenesis, tumor progression, and patient outcome.

## References

[1] Semenza GL (2012). Hypoxia-inducible factors: mediators of cancer progression and targets for cancer therapy. Trends Pharmacol Sci.

[2] Kondo S, Mukudai Y, Soga D, Nishida T, Takigawa M, Shirota T (2014). Differential expression of vascular endothelial growth factor in high- and low-metastasis cell lines of salivary gland adenoid cystic carcinoma. Anticancer Res.

[3] Lan L, Luo Y, Cui D, Shi BY, Deng W, Huo LL (2013). Epithelial-mesenchymal transition triggers cancer stem cell generation in human thyroid cancer cells. Int J Oncol.

[4] Jing SW, Wang YD, Chen LQ, Sang MX, Zheng MM, Sun GG (2013). Hypoxia suppresses E-cadherin and enhances matrix metalloproteinase-2 expression favoring esophageal carcinoma migration and invasion via hypoxia inducible factor-1 alpha activation. Dis Esophagus.

[5] Eckert AW, Kappler M, Schubert J, Taubert H (2012). Correlation of expression of hypoxia-related proteins with prognosis in oral squamous cell carcinoma patients. Oral Maxillofac Surg.

[6] Thorne JL, Campbell MJ (2014). Nuclear receptors and the Warburg effect in cancer. Int J Cancer.

[7] Mueckler M, Thorens B (2013). The SLC2 (GLUT) family of membrane transporters. Mol Aspects Med.

[8] Wu XH, Chen SP, Mao JY, Ji XX, Yao HT, Zhou SH (2013). Expression and significance of hypoxia-inducible factor-1α and glucose transporter-1 in laryngeal carcinoma. Oncol Lett.

[9] Rashmi R, DeSelm C, Helms C, Bowcock A, Rogers BE, Rader J (2014). AKT inhibitors promote cell death in cervical cancer through disruption of mTOR signaling and glucose uptake. PLoS One.

[10] Cui Y, Nadiminty N, Liu C, Lou W, Schwartz CT, Gao AC (2014). Upregulation of glucose metabolism by NF-κB2/p52 mediates enzalutamide resistance in castration-resistant prostate cancer cells. Endocr Relat Cancer.

[11] Zhang C, Liu J, Liang Y, Wu R, Zhao Y, Hong X (2013). Tumour-associated mutant p53 drives the Warburg effect. Nat Commun.

[12] Xu YY, Bao YY, Zhou SH, Fan J (2012). Effect on the expression of MMP-2, MT-MMP in laryngeal carcinoma Hep-2 cell line by antisense glucose transporter-1. Arch Med Res.

[13] Sun XP, Dong X, Lin L, Jiang X, Wei Z, Zhai B (2014). Up-regulation of survivin by AKT and hypoxia-inducible factor 1α contributes to cisplatin resistance in gastriccancer. FEBS J.

[14] Zhang L, Huang G, Li X, Zhang Y, Jiang Y, Shen J (2013). Hypoxia induces epithelial-mesenchymal transition via activation of SNAI1 by hypoxia-inducible factor-1α in hepatocellular carcinoma. BMC Cancer.

[15] Chen XH, Bao YY, Zhou SH, Wang QY, Wei Y, Fan J (2013). Glucose transporter-1 expression in CD133+ laryngeal carcinoma Hep-2 cells. Mol Med Rep.

[16] Kobayashi M, Kaida H, Kawahara A, Hattori S, Kurata S, Hayakawa M (2012). The relationship between GLUT-1 and vascular endothelial growth factor expression and 18F-FDG uptake in esophageal squamous cell cancer patients. Clin Nucl Med.

[17] Eckert AW, Lautner MH, Schütze A, Taubert H, Schubert J, Bilkenroth U (2011). Coexpression of hypoxia-inducible factor-1α and glucose transporter-1 is associated with poor prognosis in oral squamous cell carcinoma patients. Histopathology.

[18] Ayala FR, Rocha RM, Carvalho KC, Carvalho AL, da Cunha IW, Lourenço SV (2010). GLUT1 and GLUT3 as potential prognostic markers for oral squamous cell carcinoma. Molecules.

[19] Demasi AP, Costa AF, Altemani A, Furuse C, Araújo NS, Araújo VC (2010). Glucose transporter protein 1 expression in mucoepidermoid carcinoma of salivary gland: correlation with grade of malignancy. Int J Exp Pathol.

[20] Ohba S, Fujii H, Ito S, Fujimaki M, Matsumoto F, Furukawa M (2010). Overexpression of GLUT-1 in the invasion front is associated with depth of oral squamous cell carcinoma and prognosis. J Oral Pathol Med.

[21] Ciampi R, Vivaldi A, Romei C, Del Guerra A, Salvadori P, Cosci B (2008). Expression analysis of facilitative glucose transporters (GLUTs) in human thyroid carcinoma cell lines and primary tumors. Mol Cell Endocrinol.

[22] Zhou S, Wang S, Wu Q, Fan J, Wang Q (2008). Expression of glucose transporter-1 and -3 in the head and neck carcinoma—the correlation of the expression with the biological behaviors. ORL J Otorhinolaryngol Relat Spec.

[23] Kaida H, Hiromatsu Y, Kurata S, Kawahara A, Hattori S, Taira T (2011). Relationship between clinicopathological factors and fluorine-18-fluorodeoxyglucose uptake in patients with papillary thyroid cancer. Nucl Med Commun.

[24] Kaida H, Ishibashi M, Yuzuriha M, Kurata S, Arikawa S, Kawahara A (2010). Glucose transporter expression of an esophageal gastrointestinal tumor detected by F-18 FDG PET/CT. Clin Nucl Med.

[25] Müssig K, Wehrmann T, Dittmann H, Wehrmann M, Ueberberg B, Schulz S (2012). Expression of the proliferation marker Ki-67 associates with tumour staging and clinical outcome in differentiated thyroid carcinomas. Clin Endocrinol (Oxf).

[26] Kondo Y, Yoshikawa K, Omura Y, Shinohara A, Kazaoka Y, Sano J (2011). Oncol Clinicopathological significance of carbonic anhydrase 9, glucose transporter-1, Ki-67 and p53 expression in oral squamous cell carcinoma. Oncol Rep.

[27] Yan SX, Luo XM, Zhou SH, Bao YY, Fan J, Lu ZJ (2013). Effect of antisense oligodeoxynucleotides glucose transporter-1 on enhancement of radiosensitivity of laryngeal carcinoma. Int J Med Sci.

[28] Shimanishi M, Ogi K, Sogabe Y, Kaneko T, Dehari H, Miyazaki A (2013). Silencing of GLUT-1 inhibits sensitization of oral cancer cells to cisplatin during hypoxia. J Oral Pathol Med.

[29] Ping W, Sun W, Zu Y, Chen W, Fu X (2014). Clinicopathological and prognostic significance of hypoxia-inducible factor-1α in esophageal squamous cell carcinoma: a meta-analysis. Tumour Biol.

[30] Koperek O, Akin E, Asari R, Niederle B, Neuhold N (2013). Expression of hypoxia-inducible factor 1 alpha in papillary thyroid carcinoma is associated with desmoplastic stromal reaction and lymph node metastasis. Virchows Arch.

[31] Li DW, Zhou L, Jin B, Xie J, Dong P (2013). Expression and significance of hypoxia-inducible factor-1α and survivin in laryngeal carcinoma tissue and cells. Otolaryngol Head Neck Surg.

[32] Douglas CM, Bernstein JM, Ormston VE, Hall RC, Merve A, Swindell R (2013). Lack of prognostic effect of carbonic anhydrase-9, hypoxia inducible factor-1α and bcl-2 in 286 patients with early squamous cell carcinoma of the glottic larynx treated with radiotherapy. Clin Oncol (R Coll Radiol).

[33] Cabanillas R, Rodrigo JP, Secades P, Astudillo A, Nieto CS, Chiara MD (2009). The relation between hypoxia-inducible factor (HIF)-1alpha expression with p53 expression and outcome in surgically treated supraglottic laryngeal cancer. J Surg Oncol.

[34] Edge SB, Compton CC (2010). The American Joint Committee on Cancer: the 7th edition of the AJCC cancer staging manual and the future of TNM. Ann Surg Oncol.

[35] Starska K, Kulig A, Lukomski M (2006). Tumor front grading in prediction of survival and lymph node metastases in patients with laryngeal carcinoma. Adv Med Sci.

[36] Cadman E, Bostwick JR, Eichberg J (1979). Determination of protein by a modified Lowry procedure in the presence of some commonly used detergents. Anal Biochem.

[37] Kwon OJ, Park JJ, Ko GH, Seo JH, Jeong BK, Kang KM (2014). HIF-1α and CA-IX as predictors of locoregional control for determining the optimal treatment modality for early-stage laryngeal carcinoma. Head Neck.

[38] Burstein DE, Nagi C, Kohtz DS, Lumerman H, Wang BY (2006). Immunohistochemical detection of GLUT1, p63 and phosphorylated histone H1 in head and neck squamous intraepithelial neoplasia: evidence for aberrations in hypoxia-related, cell cycle- and stem-cell-regulatory pathways. Histopathology.

[39] Zhang X, Han S, Han HY, Ryu MH, Kim KY, Choi EJ (2013). Risk prediction for malignant conversion of oral epithelial dysplasia by hypoxia related protein expression. Pathology.

[40] Xie J, Li DW, Chen XW, Wang F, Dong P (2013). Expression and significance of hypoxia-inducible factor-1α and MDR1/P-glycoprotein in laryngeal carcinoma tissue and hypoxic Hep-2 cells. Oncol Lett.

[41] Fenske W, Völker HU, Adam P, Hahner S, Johanssen S, Wortmann S (2009). Glucose transporter GLUT1 expression is an stage-independent predictor of clinical outcome in adrenocortical carcinoma. Endocr Relat Cancer.

[42] Kim BW, Cho H, Chung JY, Conway C, Ylaya K, Kim JH (2013). Prognostic assessment of hypoxia and metabolic markers in cervical cancer using automated digital image analysis of immunohistochemistry. J Transl Med.

[43] Roh JL, Cho KJ, Kwon GY, Ryu CH, Chang HW, Choi SH (2009). The prognostic value of hypoxia markers in T2-staged oral tongue cancer. Oral Oncol.

[44] Schrijvers ML, van der Laan BF, de Bock GH, Pattje WJ, Mastik MF, Menkema L (2008). Overexpression of intrinsic hypoxia markers HIF1alpha and CA-IX predict for local recurrence in stage T1-T2 glottic laryngeal carcinoma treated with radiotherapy. Int J Radiat Oncol Biol Phys.

[45] Yamada T, Uchida M, Kwang-Lee K, Kitamura N, Yoshimura T, Sasabe E (2012). Correlation of metabolism/hypoxia markers and fluorodeoxyglucose uptake in oral squamous cell carcinomas. Oral Surg Oral Med Oral Pathol Oral Radiol.

